# Impact of an Evidence-Based Bundle on Catheter-Associated Sepsis Incidence in Neonatal Intensive Care: A Quality Improvement Project

**DOI:** 10.3390/diseases13120386

**Published:** 2025-11-28

**Authors:** Anna Sala, Valentina Pivetti, Francesca Castoldi, Francesca Viaroli, Marco Chiera, Gianluca Lista, Francesco Cavigioli

**Affiliations:** 1 Department of Neonatology and Neonatal Intensive Care Unit, V. Buzzi Children’s Hospital, 20154 Milan, Italyvalentina.pivetti@asst-fbf-sacco.it (V.P.); francesca.castoldi@asst-fbf-sacco.it (F.C.); francesca.viaroli@asst-fbf-sacco.it (F.V.); gianluca.lista@gmail.com (G.L.); 2 Foundation Centre, Osteopathic Medicine (COME) Collaboration, 65121 Pescara, Italy; marco.chiera.90@gmail.com

**Keywords:** CLABSI, NICU, CVC, bundle, infants, CoNS

## Abstract

**Background:** Central line-associated bloodstream infections (CLABSIs) in neonatal intensive care units (NICUs) pose a significant risk, especially for very low birth weight infants due to their immature immune systems and the need for invasive procedures. The implementation of evidence-based bundles, as recommended by international guidelines, has proven effective in significantly reducing CLABSI rates, improving clinical outcomes, and lowering hospital costs. However, evidence from long-term, real-world quality-improvement programs in European NICUs—especially those using repeated PDSA cycles and detailed monitoring across multiple periods—remains limited. **Methods:** This quality improvement prospective study, conducted in the NICU of “V. Buzzi” Children’s Hospital, aimed to reduce high CLABSI rates using a plan-do-study-act (PDSA) framework. A multidisciplinary team developed and implemented a new evidence-based central line bundle in 2021, focusing on standardized practices, enhanced training, and monitoring. The study analyzed 594 CVCs placed in 348 neonates across a total 4-years period (P1–P12). **Results:** Implementation of a central line bundle significantly reduced CLABSI rates from 29.1 to 2.2 per 1000 CVC days (*p*-value 0.002), with notable variations during intermediate periods. Birth weight and study period progression were the only variables significantly associated with CLABSI reduction. **Conclusions:** Infection rates dropped significantly post-intervention, achieving zero in one of the latest periods: continuous monitoring, staff training, and targeted interventions were pivotal. Future efforts will focus on refining practices, increasing tunneled centrally inserted central catheter (CICC) use, and sustaining prevention measures.

## 1. Introduction

Healthcare-associated infections (HAIs) are among the most common complications in neonatal intensive care units (NICUs), especially in very low birth weight (VLBW) infants. Due to their immunological immaturity and frequent need for invasive procedures, these infants are at particularly high risk of infection [1].

Among HAIs, central line-associated bloodstream infections (CLABSIs) are a major cause of morbidity, mortality, and increased healthcare costs in NICUs [2]. The most common causative pathogens are Gram-positive bacteria—particularly coagulase-negative staphylococci—[3] followed by Gram-negative bacteria and fungi [4]. Risk factors for CLABSI include transcutaneous pathogen migration, contamination during catheter insertion or handling, and hematogenous spread from distant infections [3,5]. Intrinsic risks include neonatal immune immaturity and compromised skin barrier; extrinsic factors include catheter material, prolonged use, and parenteral nutrition [6].

Over the past two decades, the introduction of evidence-based “bundles” has proven to be an effective strategy to reduce CLABSI incidence in NICUs worldwide [4,7,8,9,10]. These bundles typically include evidence-based items selected and proposed in international guidelines and recommendations, such as strict hand hygiene, full barrier precautions, antiseptic skin preparation, careful catheter site selection, secure fixation, and prompt removal when no longer needed [6,11]. Studies consistently show that bundle implementation, combined with continuous education and feedback to healthcare staff, can lead to a marked reduction in infection rates [12,13].

Despite this evidence, data from our national context remain limited, and local adherence to preventive measures may vary over time. To address this gap, we developed and implemented a targeted CLABSI prevention bundle in our NICU, coupled with multidisciplinary educational interventions.

## 2. Materials and Methods

### 2.1. Study Design and Population

This quality improvement study, based on the Plan-Do-Study-Act (PDSA) framework, was conducted prospectively in the NICU of “Vittore Buzzi” Children’s Hospital in Milan, Italy, a tertiary-level unit with 20 beds. The study aimed to address a perceived high CLABSI rate. The Plan–Do–Study–Act (PDSA) framework was selected because it is widely recommended for healthcare quality-improvement initiatives and allows iterative testing, monitoring, and refinement of clinical practices, making it particularly suitable for implementing and evaluating changes in central line care.

In January 2021, a multidisciplinary improvement team was formed to analyze existing practices, identify key issues such as lack of standardization, absence of a central line monitoring system, and insufficient staff training, and develop an evidence-based central line bundle. Based on the knowledge acquired from the literature and the availability of new materials, the bundle included the following items:

- Perform meticulous mapping of all superficial veins following the RaSuVA (Rapid Superficial Vein Assessment) protocol [14].
- Estimate the required catheter length to reach the superior or inferior vena cava.
- Proper hand hygiene.
- Use full barrier precautions, including sterile gloves, sterile gown, non-sterile cap, non-sterile mask, and comprehensive sterile draping of the patient.
- Perform skin antisepsis using single-use sterile predosed applicators with 2% chlorhexidine in an alcoholic solution (70% isopropyl alcohol), applying the minimum effective dose for the shortest necessary time [15,16].
- Achieve maximum stabilization of the venous access to prevent dislodgment, minimize catheter movement at the exit site, minimizing further manipulations at the insertion site and reducing the risk of infection and thrombosis. The stabilization strategies include:
  - Cyanoacrylate glue: seals the exit site to prevent bleeding, protects the catheter from extraluminal contamination, and stabilizes it effectively [17,18,19]. It is a safe tool with no contraindications, providing long-lasting effects (up to one week). It can be easily removed with saline solution.
  - Sutureless fixation device: Provides secure and atraumatic stabilization.
  - Transparent, semipermeable, high-breathability polyurethane dressing: fully isolates the catheter’s exit site, serving as a mechanical barrier while allowing direct visualization [20]. Its high breathability ensures adequate skin ventilation, preventing maceration.
- The indication for CVC use should be reassessed daily, and the catheter should be removed within 24 h if no longer deemed necessary.
- The insertion site should be inspected daily for signs of infection; if infection is suspected, the CVC should be removed within 24 h.

In September 2021, the newly developed bundle was implemented following comprehensive training sessions—both theoretical and practical—for all NICU staff. The implementation focused on ensuring adherence to the updated protocol during CVC insertion and management. Daily monitoring of compliance with the bundle began immediately after its introduction. Data on all CVC insertions, complications, and outcomes were systematically recorded in a dedicated database.

**Figure 1:**
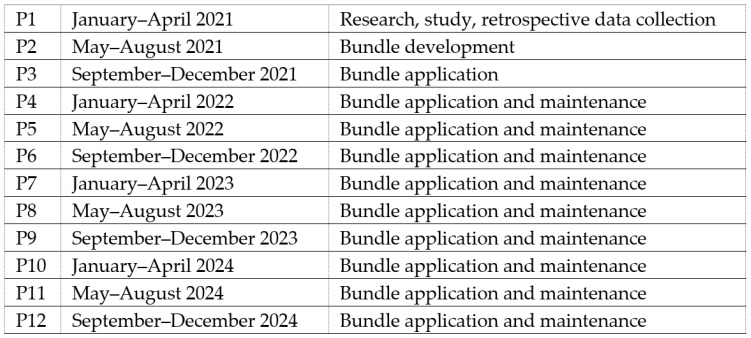
Description of study periods.

The study population included neonates requiring central venous catheters (CVCs) between January 2021 and December 2024, excluding patients transferred with pre-existing CVCs. The study period was divided into 12 consecutive periods of four months each: pre-intervention (P1—research, study, retrospective data collection), intervention (P2—bundle development and initial application), immediate post-intervention (P3—bundle application), and maintenance phases (P4–P12—bundle application, periodic data feedback to NICU staff) (Figure 1). According to the PDSA methodology, results were evaluated and analyzed on an ongoing basis and corrective measures were implemented when the CLABSI rate showed worsening [21].

CLABSI was defined according to CDC/NHSN criteria as a laboratory-confirmed bloodstream infection in a patient with a central line in place for more than 2 days, with no other identifiable source of infection [22]. Blood cultures were analyzed in the Luigi Sacco Hospital microbiology laboratory as per regular practice. Pathogens were identified using automated culture systems, with species confirmation performed through standard biochemical or molecular methods and antibiograms drafted according to the most recent EUCAST breakpoint tables.

### 2.2. Outcomes

Results were finally analyzed to assess the primary endpoint, which was the reduction in CLABSI rates (calculated per 1000 catheter days) by comparing pre-intervention (P1) and subsequent periods (P2–P12). Secondary endpoints examined additional influencing variables such as gestational age, birth weight, catheter type, and dwell time.

### 2.3. Sample Size

The sample size was calculated using G*Power 3.1.9.4, assuming a medium effect size (w = 0.3) based on Cohen’s guidelines and previous studies reporting significant reductions in CLABSI rates with structured bundles [7,8]. Considering α = 0.05, a power of 0.95, and 12 groups (P1–P12, each representing a 4-month period), the calculated sample size was 280, which was increased to 308 to account for an anticipated 10% dropout.

### 2.4. Statistical Analysis

Descriptive and inferential statistical analyses, including z-tests, linear regression, and ANOVA, were used to evaluate the impact of the intervention and guide ongoing quality improvement efforts, using R analysis software, version 3.6.1, within the RStudio development environment, version 1.4.1103.

Potential confounding factors were identified a priori based on clinical relevance and previous literature. These included birth weight, gestational age at birth, type of CVC (ECC vs. CICC), catheter dwell time, and days of life at CVC placement. All variables were included in multivariable linear regression models to adjust for their potential influence on CLABSI rates. Stepwise regression was subsequently performed to identify the most relevant predictors.

Data completeness was verified prior to analysis; no missing data were identified. Had missing data been present, appropriate methods such as multiple imputation or full information maximum likelihood would have been applied [23].

### 2.5. Ethical Considerations

The study was conducted in accordance with the Declaration of Helsinki and was approved by the Ethics Committee ‘Comitato Etico Territoriale Lombardia 1’ (protocol code: CLABSI1; opinion no. CET 155-2025, 3 July 2025).

## 3. Results

### 3.1. Description of the Population

The total number of CVCs considered during the study period was 594, placed in a total population of 348 patients, distributed variably across different periods, with a minimum of 35 CVCs (P8) and a maximum of 61 (P3). The table below (Table 1) provides a description of the demographic and clinical characteristics of the neonates who received a CVC across the 12 periods (P1 to P12), detailing sex, gestational age (GA), the percentage of neonates with gestational age under 32 weeks, and birth weight stratified into different weight categories.

The sex distribution was balanced between males (M) and females (F) in each period, with a slight predominance of males in all periods except P2, P9, and P11. The mean gestational age ranged from a minimum of 30 weeks (P1) to a maximum of 35 + 3 weeks (P8). The proportion of CVCs placed in neonates with a gestational age under 32 weeks varied across periods, with P1 (pre-intervention) having the highest percentage (80%) and P8 the lowest (17%), and the mean birth weight ranged from a minimum of 1339 g (P1) to 2203 g (P8). The distribution by weight categories highlighted a presence of extremely low birth weight (ELBW, <1000 g) neonates of 54% during P1, whereas in P8, the majority of CVCs were placed in neonates with a birth weight over 2500 g (46%).

We conducted a one-way ANOVA analysis to identify potential differences in birth weights among neonates in the different periods. Birth weights from the 12 were compared. The one-way ANOVA revealed statistically significant differences in birth weights across periods (*p* < 0.05). Tukey’s post hoc test identified significant differences in birth weights between periods 1 and 8, periods 1 and 11 and periods 8 and 9 (Table 2).

Similarly, a one-way ANOVA was conducted to analyze differences in gestational ages across groups. The test yielded a *p*-value < 0.05, showing significant differences in mean gestational ages among the groups. Tukey’s post hoc test was subsequently performed to compare mean gestational ages between all possible pairs of groups. The group pairs showing statistically significant differences were P1-P8, P1-P11, P8-P5, P8-P9, P8-P10 and P8-P12 (*p* < 0.05) (Table 3).

### 3.2. CLABSI Rate

**Figure 2:**
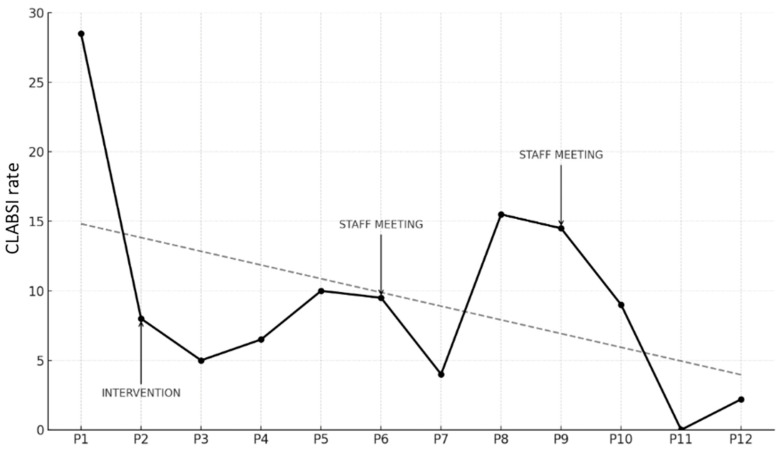
Trend of CLABSI rate (number of infections/1000 CVC days) across study periods, including timing of intervention and data feedback sessions (staff meeting). The dashed line represents the regression line illustrating the overall decreasing trend in CLABSI rates across the study period.

Trends in CLABSI Rates (expressed as infections per 1000 CVC days) are illustrated in the graph below (Figure 2).

The starting point was a CLABSI rate in P1 of 29.1 per 1000 CVC days. During P2, when the bundle was introduced, a reduction was immediately observed, with the rate dropping to 7.7. This trend continued in P3, reaching 5.2. In P4, there was a slight increase to 7.0, followed by a further rise in P5 to 10.1. Due to the persistence of higher rates compared to earlier periods, feedback sessions were conducted with the entire medical and nursing staff, reviewing the rates and providing updates on the bundle and related practices, with the aim of re-emphasizing the relevance of the issue, assuming a decline in adherence to the bundle due to reduced staff attention, the goal was to restore a high level of awareness regarding the procedures.

In P6, a slight reduction was observed, with the rate decreasing to 9.8, and in P7, a further reduction to 3.7 infections per 1000 CVC days was achieved. However, P8 showed a significant increase to 15.8, which remained high at 14.5 in P9. Consequently, another feedback session was held, this time exclusively with medical staff, focusing on data review and revising the CVC insertion procedure, with an emphasis on epicutaneo–caval catheters (ECCs), which were more commonly used. By P10 the rate was stable at 9.3 infections per 1000 CVC days. Finally, in P11, a decline to 0 infections was achieved, followed by a slight increase in the last period (2.2 infections per 1000 CVC days).

When comparing the pre-intervention period (P1) to all subsequent periods using a test of proportion comparisons, the reduction in CLABSI rates was found to be statistically significant in all periods except P8 and P9 (Table 4).

**Figure 3:**
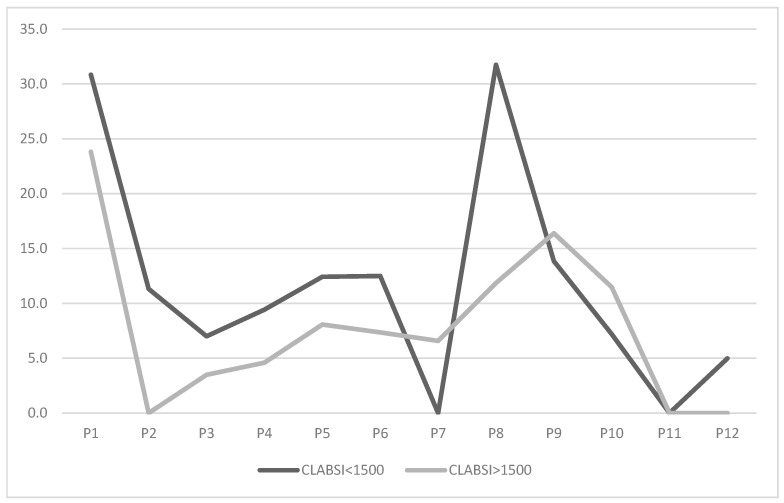
Trend of CLABSI rate (number of infections/1000 CVC days) across study periods, stratified by neonatal population with between <1500 g and >1500 g.

Given the higher infection risk among very low birth weight (VLBW) neonates, we stratified the population into neonates with a birth weight <1500 g and >1500 g. Results are presented in the graph below (Figure 3).

CLABSI rates for neonates < 1500 g were initially higher (30.8) compared to those > 1500 g (23.8). After bundle implementation, rates for neonates < 1500 g dropped to 11.3 in P2. Their trend mirrored the overall rate, reaching a minimum of 0 in P7 before rising in P8 to levels comparable to pre-intervention, and then to 0 in P11. Neonates > 1500 g showed a reduction to 0 as early as P2, with variations aligning with the overall trend, culminating at a stable 0 in P11 and P12.

To identify additional variables associated with the reduction in CLABSI rates over time, we first used linear regression. Variables considered included: progression of periods (measured as a discrete variable), birth weight (in hectograms), weight at the time of CVC placement (in grams), gestational age at birth, CVC type, catheter dwell time, and days of life at CVC placement. Among these variables, only progression of periods was statistically significant (*p* < 0.001), indicating that higher values of the temporal period were associated with a decrease in the outcome variable. All other predictors, including birth weight, gestational age, and catheter-related variables, were not statistically significant in this global model.

Stepwise regression was performed to select the most relevant predictors. Across all models, progression of periods was consistently retained and statistically significant. Birth weight was included in some models, showing a modest association in certain iterations. Other variables, including catheter dwell time and days of life at CVC placement, were occasionally retained but did not reach statistical significance.

The final model identified period progression and birth weight as significant predictors (Table 5).

### 3.3. Usage and Type of Catheter

**Figure 4:**
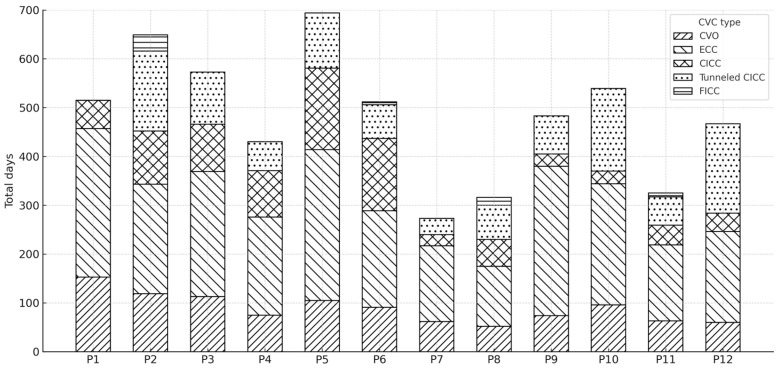
Distribution of total central venous catheter (CVC) days by catheter type across study periods (P1–P12).

Data regarding the use of different types of central vascular devices (umbilical venous catheter–UVC, epicutaneo–caval catheter–ECC, centrally inserted central catheter–CICC, tunneled CICC, and femorally inserted central catheter–FICC) across the various study periods (P1–P11) are shown in Figure 4.

The cumulative number of CVC days for each period was stratified by catheter type, with each bar representing the total CVC days per period and the stacked segments indicating the contribution of each catheter type.

### 3.4. Microbiological Aspects

In our population, coagulase-negative staphylococci were the leading cause of CLABSI, accounting for 73% of cases, with *Staphylococcus capitis* being the most frequent (25%), followed by *S. haemolyticus* (21%), *S. epidermidis* (18%), and *S. hominis* (9%). *Staphylococcus aureus* caused 7% of infections. Gram-negative bacteria (*Escherichia coli*, *Klebsiella pneumoniae*, *Serratia marcescens*) accounted for 6% of cases, including one instance of co-infection with *S. capitis*. *Enterococcus faecalis* was identified in 2% of cases and was also involved in co-infections with Gram-negative organisms. Fungal infections represented 6% of CLABSI, involving three *Candida* species (*C. albicans*, *C. pelliculosa*, *C. parapsilosis*), one of which was isolated alongside a coagulase-negative staphylococcus.

## 4. Discussion

The aim of our study was to evaluate the effectiveness of a new bundle for the insertion and management of CVCs in reducing CLABSI in our NICU. CLABSIs represent a significant cause of morbidity and mortality, especially among VLBW neonates, and are among the most lethal preventable infections in healthcare settings [24]. Given the high prevalence of preterm and VLBW infants in our unit, an optimal approach to the management of central venous access was crucial for improving the quality of care.

The primary endpoint was to assess how the introduction of the new practices described in the bundle influenced the infection rate between P1 (pre-intervention, plan phase of PDSA cycle) and the subsequent post-intervention periods (do phase) while constantly revising the data (study phase) and therefore improving and correcting the interventions (act phase). As shown in Figure 1, the reduction in the CLABSI rate during the intervention period (P2) was remarkable and statistically significant (Table 4). Further evaluation of P3 revealed an additional statistically significant reduction.

This outcome aligns with the literature regarding the impact of implementing a dedicated bundle on CLABSI rates [25]. The heightened awareness due to the novelty and recent introduction of the practices among the staff had a positive effect on the rate as early as P2, even before all measures and materials were formally implemented. The pre-intervention infection rate of 29.1 CLABSIs per 1000 CVC days, while high, is comparable to similar studies. For example, the study by Sinha et al. [26] conducted in a Level III NICU in London reported a higher pre-intervention rate of 31.6, while Resende et al. observed an initial rate of 24.1 infections per 1000 CVC days, slightly lower than the rate observed in our unit [27].

The rate trend during the maintenance periods (P4–P12) showed two main increases: the first in P5 (10.1) and the second, more significant, in P8 (15.8). During this second increase, the statistical significance of the reduction compared to the pre-intervention rate was lost. In both cases, data collection and analysis by our quality improvement team identified the upward trend. Considering the persistent deterioration in CLABSI rates, data review and staff updates were organized in September 2022 and January 2024 (Figure 1). Both interventions resulted in a subsequent decrease in CLABSI rates, highlighting the critical role of data collection, monitoring, and analysis in quality improvement initiatives, as recognized in the literature [12,28].

Achieving a zero-infection rate during one of the final study periods represents a major success for the infection prevention and control measures codified in the bundle and implemented over time. This result is clinically significant as it demonstrates not only the effectiveness of the initial intervention but also the importance of continuous monitoring and reinforcement of the introduced practices. Specifically, implementation of the bundle reduced CLABSI rates from 29.1 to 2.2 per 1000 CVC days, corresponding to a 92% reduction. The effect was observed across all weight strata, including neonates < 1500 g, highlighting a substantial clinical and economic impact. These efforts impact patient safety and economic factors such as hospitalization costs. It is well established that the incidence of infections in preterm infants significantly affects both mortality and morbidity. Persistent inflammatory immune responses from sepsis contribute to the multifactorial pathogenesis of prematurity-related conditions (e.g., bronchopulmonary dysplasia, necrotizing enterocolitis, intraventricular hemorrhage, periventricular leukomalacia), increasing their incidence [29]. Additionally, the morbidity associated with infectious events extends hospital stays and costs [2]. Therefore, our results are highly relevant for the broad range of benefits they provide, not limited to the clinical sphere.

It is equally important to note that the achievement of a zero CLABSI rate was immediately followed by a slight increase in the subsequent period; therefore, ongoing data collection, vigilance, and continuous staff education are essential to sustain these results over time.

When stratifying the population by birth weight, we re-evaluated trends focusing on VLBW neonates (Figure 2). The rate trend closely mirrored the overall trend, though higher values were observed during the first six periods (P1–P6). This finding is consistent with the increased risk of nosocomial infections and late-onset sepsis (LOS) in the VLBW category, as reported in the literature [1,30,31,32]. It is also noteworthy that during P7 and P10, the rate for this subpopulation was lower than the overall rate (0 vs. 3.7 in P7, 7.2 vs. 9.3 in P10). These periods correspond to the drops following a peak, immediately after feedback and staff update sessions. This suggests that heightened awareness among staff particularly impacts this vulnerable patient group, who is known and perceived as being most susceptible to such complications.

The influence of birth weight on infection rates was also confirmed through stepwise regression analyses. The final proposed model identified birth weight as a significant variable, with a small negative effect, suggesting that neonates with higher birth weight tend to have a lower risk of infection.

The other variable found to be influential in the final stepwise regression model was the period, considered as a discrete numerical variable (P1–P12), with a negative coefficient. This result suggests that, as time progresses, there is a general trend toward decreasing infection rates, regardless of the fluctuations observed in the specific data for each period. This may reflect a progressive improvement in the adoption and effectiveness of infection prevention and control practices implemented over the various periods.

Other variables, such as gestational age, catheter type, and the number of days of life at the time of catheter placement, were not significant and were eliminated from the stepwise model, indicating that their impact on infection rates was not relevant in the context of our study.

Regarding the different types of central venous catheters, it is important to highlight the role of tunneled CICCs in specific critical patients, helping reduce infectious complications due to the tunneling procedure, which allows for a well-reasoned and optimal choice of the exit site [11]. However, the placement of tunneled CICC in the neonatal period remains a skill requiring dedicated training and practice, which few professionals currently possess. As a result, in many units, including ours, the use of tunneled CICC is quite limited, sometimes favoring non-tunneled CICC. However, non-tunneled devices should be utilized solely in emergency situations and retained for the minimal duration necessary [33].

The limited use of this type of CVC may have influenced the results obtained. Further studies would be necessary to evaluate whether a more extensive use of these highly effective devices, which are increasingly being adopted in neonatal care, could significantly impact CLABSI rates in neonatal intensive care units.

The variable catheter dwell time was also not statistically significant in influencing infection rates over time in the stepwise regression analysis. This finding contrasts with, identifying CVC dwell time as a risk factor for the development of CLABSI [32,34,35]. The reasons for our result could be attributed to two elements. The first element is the differing average dwell times specific to each type of CVC. In fact, while an UVC can remain in place for a maximum of 7 days [33], an ECC has a dwell time that should not exceed 2–3 weeks [36]. Non-tunneled CICC and FICC catheters should be used for short periods, whereas tunneled catheters are considered long-term CVCs. This inherent disparity in dwell time across different CVC types may have negatively influenced the analysis. The second element to consider relates to the frequent removal of infected CVCs. Although removal is not always performed due to difficulties in securing alternative access or the possibility of conservative treatment of the infection, removal upon detecting CLABSI may have influenced our data by reducing the dwell time of those infected catheters.

The ANOVA analyses performed to compare gestational age and birth weight across the study periods (Table 2 and Table 3) revealed statistically significant differences between certain periods. These findings are highly relevant: in particular, the substantial differences between P1 and P8, with neonates in P1 showing significantly lower gestational age and birth weight, help explain the high infection rate observed in P1. Conversely, they make the increase observed in P8 even more concerning, as the population in this period, given its characteristics, should have been less predisposed to infectious complications. Another noteworthy finding is the significant difference between P1 and P11, again with lower gestational ages and birth weights in P1. The achievement of zero CLABSI in P11, compared with the initial rate of 29.1 in P1, should therefore be interpreted in light of the significant differences between the two populations, which may have influenced the attainment of this remarkable outcome.

From a microbiological perspective, the data emerging from our study are consistent with what has been reported in the literature: CoNS, such as *Staphylococcus capitis*, are the microorganisms most frequently involved in these infections in preterm and VLBW neonates, reported as the cause of over 50% of CLABSIs, particularly in the latter category [3,37]. In particular, *S. capitis* is increasingly emerging as a predominant pathogen due to specific microbiological characteristics that make it particularly persistent in the neonatal intensive care environment; this aligns with the results of our study, which identifies it as the cause of 25% of infections [38,39]. The distribution of pathogens causing CLABSI remained unchanged before and after the intervention, suggesting that the observed reduction in infections was not related to changes in microbial etiology.

The limitations of our study include significant population differences across study periods, which may have influenced the outcomes, and the observational nature of the study, leaving room for potential uncontrolled confounders. Furthermore, this study was conducted at a single center, which may limit the generalizability of the findings to other facilities with different patient populations, clinical conditions, or staff training levels. Future multicenter studies are needed to confirm and extend these results.

## 5. Conclusions

Our study highlighted the importance of implementing a bundle for the insertion and management of central venous catheters (CVCs) in neonatal intensive care units (NICUs) to prevent central line-associated bloodstream infections (CLABSIs). The results demonstrated a significant reduction in CLABSI rates following the introduction of new practices, aligning with evidence from the literature on the effectiveness of targeted infection control strategies in this vulnerable setting.

Continuous data monitoring and analysis were crucial in identifying trends and implementing timely interventions to influence infection rates. Ongoing education and training of healthcare personnel remain essential to prevention strategies, as increased awareness among the care team was shown to positively impact infection rates.

The encouraging results achieved in the final study period, with an infection rate of zero, represent a significant milestone for patient safety and care quality in NICUs. However, sustained efforts are necessary to monitor infection trends and ensure the appropriate use of CVCs.

Future efforts will include ongoing data collection and monitoring, with regular feedback and updates for healthcare staff, incorporating innovations identified in the recent literature. A key objective is to increase the use of tunneled CICC in eligible patients, supported by the training of a department physician in this specialized skill.

In conclusion, our findings support the adoption of a systematic bundle of evidence-based practices for CLABSI prevention in neonatal care. This approach significantly improves clinical outcomes, potentially reducing morbidity and mortality in this patient population.

## Figures and Tables

**Table 1:** Description of the demographic characteristics (sex, GA, BW) of neonates with CVCs across different periods. Numbers and percentages refer to CVCs placed in patients with these characteristics. GA gestational age, SD standard deviation.

	P1	P2	P3	P4	P5	P6	P7	P8	P9	P10	P11	P12
n. catheters	59	55	61	45	59	43	40	35	52	56	43	46
Sex, n (%)												
M	33 (56)	26 (47)	33 (54)	32 (71)	31 (53)	24 (56)	22 (55)	23 (66)	25 (48)	34 (61)	20 (47)	25 (54)
F	26 (44)	29 (53)	28 (46)	13 (29)	28 (47)	19 (44)	18 (45)	12 (34)	27 (52)	22 (39)	23 (53)	21 (46)
GA (weeks), mean ± SD	30 ± 4.8	32.7 ± 4.4	32.6 ± 4.4	32.5 ± 4.8	32.3 ± 5	32.1 ± 5.5	32.7 ± 4.7	35.5 ± 3.5	31.3 ± 4.5	31.8 ± 4.9	34 ± 4.5	32 ± 4.7
Neonates with GA < 32 weeks, n (%)	47 (80)	22 (40)	30 (49)	24 (53)	31 (53)	23 (53)	21 (53)	6 (17)	35 (67)	28 (50)	19 (44)	24 (52)
Birth weight (g), mean ± SD	1339 ± 837	1673 ± 857	1733 ± 884	1817 ± 936	1759 ± 1018	1881 ± 1004	1875 ± 923	2203 ± 924	1528 ± 874	1716 ± 983	1993 ± 1007	1730 ± 981

**Table 2:** Results of Tukey’s multiple comparison test for birth weight comparison across different periods. CI Confidence Interval.

Pair	Difference	Lower CI	Upper CI	*p*-Value
P8-P1	864.817	210.770	1518.865	0.001
P11-P1	654.056	39.382	1268.730	0.026
P9-P8	−675.362	−1345.600	−5.124	0.046

**Table 3:** Results of the Analysis of Variance (ANOVA) and Tukey’s multiple comparison test for gestational age comparison across different periods. SE Standard Error, CI Confidence Interval.

Pair	Difference	Lower CI	Upper CI	*p*-Value
P8-P1	11.553	4.972	18.133	0.000
P11-P1	8.269	2.084	14.453	0.001
P8-P5	6.841	0.260	13.421	0.033
P9-P8	−8.708	−15.451	−1.964	0.002
P10-P8	−7.579	−14.225	−0.933	0.011
P12-P8	−6.965	−13.884	−0.047	0.047

**Table 4:** Number of infections, total CVC days, and CLABSI rate across periods, with *p*-value from proportion comparison test between P1 and subsequent periods (P2–P11). Italicized values indicate statistically significant *p*-values.

	n. CLABSI	CVC Days	CLABSI/1000 CVC Days	*p*-Value
P1	15	515	29.1	
P2	5	649	7.7	*0.016*
P3	3	573	5.2	*0.004*
P4	3	430	7.0	*0.025*
P5	7	694	10.1	*0.026*
P6	5	512	9.8	*0.043*
P7	2	273	3.7	*0.032*
P8	5	316	15.8	0.326
P9	7	483	14.5	0.175
P10	5	539	9.3	*0.033*
P11	0	325	0.0	*0.005*
P12	1	467	2.2	*0.002*

**Table 5:** Stepwise regression model identifying variables significantly associated with CLABSI rate.

Variable	Estimate (ß)	IC 95%	*p*-Value
Intercept	17.459	15.800–19.118	<0.001
Period	−0.947	−1.129–−0.766	<0.001
Birth weight	−0.076	−0.138–−0.014	0.016

## Data Availability

The data presented in this study are available upon request from the corresponding author. The data are not publicly available due to privacy concerns.
